# Sporadic, Nontrauma-Related, Desmoid Tumor of the Pancreas: A Rare Disease—Case Report and Literature Review

**DOI:** 10.1155/2010/272760

**Published:** 2010-03-14

**Authors:** F. Polistina, G. Costantin, E. D'Amore, G. Ambrosino

**Affiliations:** ^1^Emergency and Trauma Department, Dolo Hospital, Dolo, 30031 Venice, Italy; ^2^Department of General Surgery, San Bortolo Hospital, 36100 Vicenza, Italy; ^3^Department of Human Pathology, San Bortolo Hospital, 36100 Vicenza, Italy; ^4^School of General Surgery, University of Padua, 35128 Padua, Italy

## Abstract

Desmoid tumors (DTs) are neoplasms of fibroblastic origin characterized by lack of a capsule. They are nonmetastatic and locally aggressive. Intraabdominal DTs are often observed in familial adenomatous polyposis and Gardner syndrome or subsequent to localized traumatic injury. Sporadic forms are defined as nontrauma- or nongenetic-related DTs. Isolated, sporadic pancreatic DTs have been considered anecdotal, with only 9 cases described in the literature. We report the case of a 68-year-old man with a case of sporadic cystic DT localized to the pancreatic tail. The tumor was discovered incidentally during computerized tomography performed for an unrelated condition. The patient was asymptomatic; however, biopsy was performed on the clinical suspicion of cystic cancer of the pancreas. Pathology analysis showed fibroblastic proliferation, and the diagnosis of DT was confirmed by immunohistochemical staining for beta-catenin. The patient underwent resection with no further treatment and remain disease-free 60 months after surgery.

## 1. Background

Desmoid tumors (DTs); also known as *aggressive fibromatosis* or *musculo-aponeurotic fibromatosis*) are neoplasms of fibroblastic origin characterized by lack of a capsule [1]. Desmoid tumors represent approximately 0.03% of all tumors and 3% of soft tissues tumors [1]. They are nonmetastatic and locally aggressive, with a high local recurrence rate, and arise in virtually every site in the human body [2]. Intraabdominal DTs are often observed in familial adenomatous polyposis (FAP) and Gardner syndrome or subsequent to localized traumatic injury (surgical or nonsurgical) [1–3]. Sporadic forms are defined as nontrauma- or nongenetic-related DTs. Isolated, sporadic, pancreatic DTs have been considered anecdotal, with only 9 cases described previously [4]. Here we describe the case of patient with cystic DT; to our knowledge, the third reported case.

## 2. Case Report

**Figure 1:**
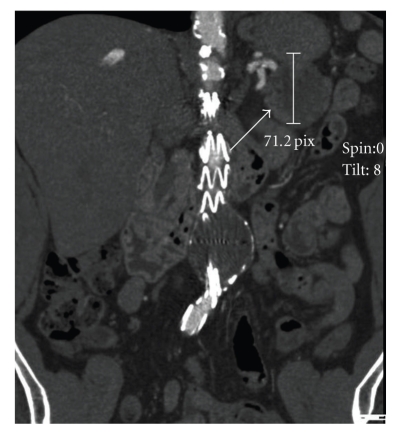
Coronal CT scan showing the tumor (arrow).

**Figure 2:**
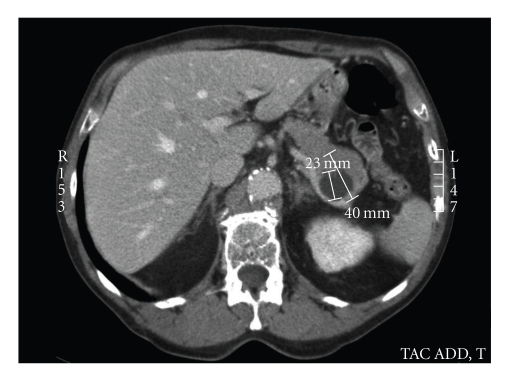
Assial CT scan showing the tumor (arrow).

A 68-year-old man presented with computerized tomography (CT) scan results showing a 5-cm solid cystic mass in the tail of the pancreas (Figures 1 and 2), with no signs of vascular or visceral invasion. There was no evidence of metastatic disease. The patient was completely asymptomatic; the CT scan was performed as part of a routine follow-up of angiographic aortic endoprosthesis placement 3 years prior for treatment of an abdominal aortic aneurysm. In addition, the patient's history included well-compensated pulmonary emphysema and a pulmonary lobectomy for a pT_2_N_0_M_0_ adenocarcinoma of the lung (lower left lobe) 5 years prior. There was no history of abdominal trauma or previous abdominal surgery and no family history to suggest genetic disease.

No clinical signs were found on physical examination. Magnetic resonance imaging failed, owing to the generation of artifacts associated with the aortic prosthesis. Levels of serum tumor markers (carcinoembryonic antigen [CEA] and CA 19.9) were in the normal range. Surgery was performed on the suspicion of cystadenocarcinoma. During the surgical exploration, the mass appeared to be localized completely to the tail of the pancreas, with no invasion of adjacent structures. There were no enlarged nodes surrounding the tumor. A left pancreatectomy was performed with spleen preservation and lymphadenectomy of the celiac axis and peripancreatic nodes. The transection margin was tumor-free. The gross appearance of the mass was of a dense grayish tumor containing a cystic cavity, with no signs of a capsule and no evidence of necrosis.

**Figure 3:**
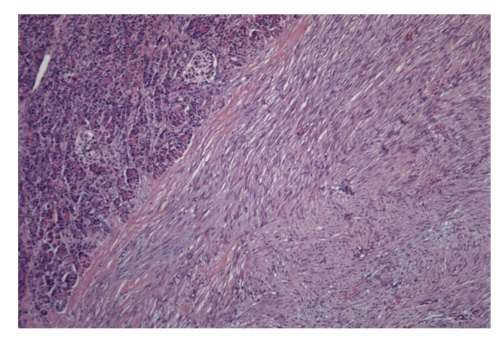
Ematossilin-eosin showing the pancreatic tissue infiltrated by the desmoid tumor.

**Figure 4:**
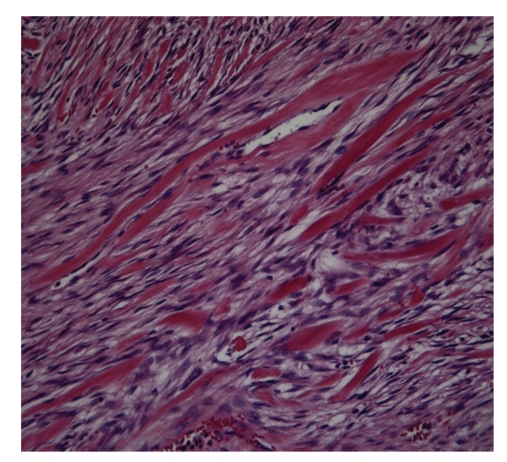
In this area the tumor shows a proliferation of spindle cells and dense collagen bundles (Ematossilin-eosin).

Histologic sections showed proliferation of spindle-shaped or stellate cells, with a fasciculate and storiform growth pattern within a background of myxoid intercellular matrix (Figure 3). Glassy, hyalinized, and keloid-like collagen fibers were also present focally. The cystic area was the result of dilatation of entrapped excretory pancreatic ducts. Immunohistochemical staining showed cytoplasmic positivity for smooth muscle actin and focal nuclear positivity for beta-catenin (Figure 4). Immunohistochemistry for desmin, CD117 (c-kit), and CD34 was negative, and the proliferation marker Ki67 stained 2% of the cells. The features were those of intraabdominal fibromatosis.

**Figure 5:**
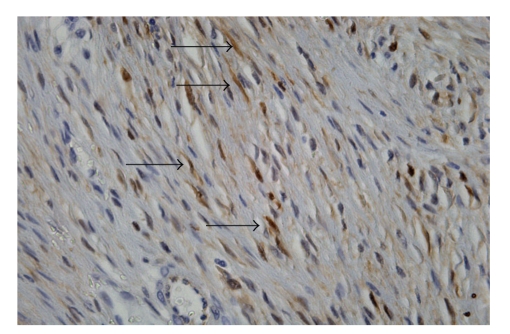
Nuclear positivity sfor B-catenin.

The tumor infiltrated the pancreas and surrounding adipose tissue. The patient developed an abscess of the pancreatic stump, which was treated by CT-guided percutaneous drainage and resolved on postoperative day 28. Oral intake was initiated on postoperative day 5, and patient was discharged on postoperative day 16. The patient is doing well and remained disease-free according to 60-month follow-up CT (Figure 5).

## 3. Discussion

Desmoid tumors are rare, benign, soft-tissue tumors characterized by fibroblastic proliferation within a collagen matrix. Recent classification includes abdominal and nonabdominal DTs; among abdominal DTs, a further distinction is made between abdominal-wall and intraabdominal DTs [5]. Intraabdominal forms are infrequent, accounting for approximately 8% of all DTs [4], and are frequently observed in FAP and Gardner syndrome. Many reports suggest that DTs occur in up to 32% of patients with FAP and in up to 29% of patients with Gardner syndrome [1, 6]. In some cases, they arise at the site of a previous unrelated operation or trauma and also occur rarely in association with pregnancy [3, 7]. Sporadic intraabdominal DTs are infrequent and appear to have a different biological behavior and clinical course [8]. Symptoms of DT are related to the local growth of the tumor and invasion of adjacent structures. The present patient was completely asymptomatic, with tumor discovery upon routine CT scan for an unrelated condition.

Pancreatic DTs are extremely rare. A review of the literature showed 9 reported cases [4, 8–14], with 2 being cystic DT [4, 13]. Only 1 case of pancreatic DT has been associated with a genetic disorder. Preoperative diagnosis of sporadic intraabdominal forms is unlikely. Some authors advocate the use of fine-needle aspiration biopsy for superficial forms [15, 16]. We decided to operate on the suspicion of cystic pancreatic cancer; the finding of DT was unexpected. Surgery is the treatment of choice for DTs, and radical resection is considered curative for all cases in which clear margins can be obtained [5, 9]. Some authors have reported successful treatment with an extended course of nonsteroidal anti-inflammatory drugs (NSAIDS) [17]. The present patient underwent left pancreatic resection with spleen preservation. Histologically, the tumor showed the typical appearance of intraabdominal fibromatosis; the mass was formed by slender fibroblasts/myofibroblasts with little cytologic atypia and low proliferative activity, separated by abundant collagen. The diagnosis was confirmed by the presence of cytoplasmic immunostaining for smooth muscle actin and nuclear staining for beta-catenin, which is usually indicative of point mutations of the *Wnt* gene and activation of the Wnt signaling pathway [18].

Diagnosis of a rare case of extraintestinal gastrointestinal stromal tumor was excluded on the bases of the morphologic findings and negative immunostaining for CD34 and CD118/c-kit. Recurrences at the site are frequent in FAP and Gardner syndrome but appear to be absent in sporadic pancreatic forms, according to the follow-up times reported in the limited literature [4, 9, 10, 13–15]. The only reported case of recurrence was in the single patient with FAP [14]. The present patient remains disease free 60 months after surgery, consistent with previous reports.

In conclusion, sporadic pancreatic DT is an extremely rare finding in common clinical practice. There are no symptoms, signs, or imaging features to aid in diagnosis. Fine-needle aspiration should be considered for incidentally discovered small lesions. However, according to current guidelines, surgery must be performed if there is any doubt as to diagnosis. Follow-up is also necessary, regardless of the low-rate of tumor recurrence.
